# Examining Boards Should Be Controlled by State Societies

**Published:** 1900-04

**Authors:** 


ARTICLE IX.

### EXAMINING BOARDS SHOULD BE CONTROLLED BY STATE SOCIETIES.

We publish elsewhere an account of a condition of affairs in connection with the California State Board of Dental Examiners, which suggests the question whether it is wise to clothe an examining board with unlimited power.

One of the first examining boards was that in the State of New York. This board was purely an examining board, being wholly without power to grant a license. It was indeed an integral part of the State Society, and upon their recommedation it was the State Society which granted diplomas to the successful candidates which gained them license to practice. Recently the dental act of New York has been altered, and the licensing power has been taken from the State Society, but the principle involved has been maintained. The Board of Examiners now report to the Board of Regents, who grant the license. This Board of Regents is a body of prominent citizens above the influences of political corruption. Being the guardians of the educational system of the State, it was deemed wise that they should control the dental schools as well.

It has long been a feature of the New York State system of government to entrust special departments of its police system to the care of associations organized with such intent. Thus we have "The Society for the Prevention of Cruelty to Children," "The Society for the Suppression of Vice," and others, all of which aim to compel obedience to the special laws within their province. In like manner the State Dental and Medical Societies not only examined candidates and granted licenses, but they likewise prosecuted illegal practitioners, the registration fees as well as the fines obtained in court being paid into the State Society's treasury. The reason for taking the licensing power away from the State

Dental Society has been given, but the examiners are still chosen by the State Society. It is thus evident that at no time have the examining boards of New York been free from the supervision of the State Dental Society, and their fine record is a strong argument against any system which allows politics to enter into the appointments, or which creates a board not controllable by the State Society.

We recently commented editorially upon the position taken by the New Jersey Board, which body declined even to make a report of its proceedings to the State Society. There was one exception among the members, Dr. Charles Meeker who claimed at that time that the best interests of the profession of his State demanded that the Dental Examiners should feel obliged to obey the wishes of the State Society.

The wisdom of his position is attested by the condition of affairs reported in California, and it is interesting to find Prof. Dunbar, in his summary of the California situation, declaring that the only remedy lies in an amendment to their law which will give the State Society control over the Board of Examiners. The charges against the California Board, the charges against the Illinois Board, the troubles with the Wisconsin Board, the declaration of independence by the New Jersey Board, all these added to the whispers against other boards, show only too clearly that the time has come for a wholesale rearrangement of the powers of dental examiners. The State laws should be amended as soon as possible, relegating the licensing power to the State Dental Societies, and restricting the duties of the Boards to examinations only.—ED "Items of Interest."

